# Morphometric evaluation of cerebral and cerebellar structures in long-term unilateral sensorineural hearing loss

**DOI:** 10.1016/j.bjorl.2025.101635

**Published:** 2025-05-08

**Authors:** Gözde Orhan Kubat, Özkan Özen, Emre Çolak

**Affiliations:** a Alanya Alaaddin Keykubat University School of Medicine, Department of Otolaryngology, Alanya, Antalya, Turkey; b Alanya Alaaddin Keykubat University School of Medicine, Department of Radiology, Alanya, Antalya, Turkey

**Keywords:** Hearing loss, Unilateral, Gray matter, Brain morphometry, Auditory cortex

## Abstract

**Objective:**

Structural changes in the brain have been identified in patients with hearing loss. However, the effect of long-term Unilateral Sensorineural Hearing Loss (USNHL) on cerebral and cerebellar morphology is not fully understood.

**Methods:**

In this study, Magnetic Resonance Imaging (MRI) of 12 long-term USNHL adults was compared with their Normal-Hearing (NH) side using Voxel-Based Morphometry (VBM). The USNHL group was also compared to a control group of 12 hearing-healthy individuals. Volume and cortical thickness changes in cerebral and cerebellar structures, possibly related to hearing loss severity and duration, were assessed using VBM.

**Results:**

The study found that, compared to the control group, USNHL patients had significantly higher measurements in the Frontal Operculum (FO) Gray Matter (GM) volume (cm^3^) (*p* = 0.024), Superior Temporal Gyrus (STG) thickness (*p* = 0.041), and cerebellum IX GM volume (cm^3^) (*p* = 0.025). No significant differences were observed between the intact and NH sides in the USNHL group (*p* > 0.05).

**Conclusion:**

Cerebral and cerebellar structures, which are essential for perceiving and interpreting sound, integrating information, and coordinating movement, may undergo morphological changes due to neuroadaptive mechanisms in long-term USNHL patients. Larger case series are needed to explore the clinical implications of these changes for diagnosis, treatment, and prognosis.

**Levels of evidence:**

Level 3.

## Highlights

• Unresolved issues remain in brain, cerebellum morphometry in USNHL patients.
• FO, STG, and lobule IX show differences between patients and control groups.
• Neurological and psychiatric disorders from damage here need hearing evaluation.
• Patients with no known causes should be checked for hearing-related changes.
• Volbrain offers fast, accurate data with systematic error correction.

## Introduction

Functional studies on SNHL have shown that the brain possesses a remarkable ability to reorganize itself in the absence of one or more sensory modalities.^1^ Studies have demonstrated that SNHL alters neural activity not only in the central auditory pathway but also in non-auditory structures such as the hippocampus, Supramarginal Gyrus (SMG), occipital gyrus, calcarine cortex, and prefrontal cortex.^1^

It is known that individuals with hearing impairment are at an increased risk of developing cognitive and emotional processing disorders and shown to impair cerebellar function.^2^

While the cerebellum is generally thought to play a role in motor control, emerging evidence suggests that it also has significant roles in sensory, cognitive, and emotional processes.^1^ Abnormal neural activity in the cerebellum has been implicated in hearing disorders, but the effects of long-term HL on cerebellar function are not fully understood.^1^

Studies on cortical reorganization in individuals with long-term unilateral SNHL have yielded mixed results. Some studies report preserved GM structure in auditory areas, while others report increased or decreased GM volume in SNHL patients.^3^

Different studies have documented experience-dependent functional brain plasticity in the primary auditory cortex of deaf individuals, and structural changes have been found outside of auditory areas as well. In fact, auditory input is critical for cognitive processing to dynamically interact with the environment. A complex combination of perceptual and higher-level cognitive factors underpins integrated auditory processing in normal-hearing individuals. Accordingly, over time, peripheral auditory deprivation, particularly chronic and sustained deprivation, may alter the auditory cortex and lead to more extensive structural reorganization beyond auditory cortical regions. Although neural plasticity resulting from auditory deprivation has been extensively studied, there is limited data on changes in both auditory and non-auditory cortical structures and their relationships with hearing ability and duration of HL in hearing-impaired individuals.^4^

In individuals with HL, the remaining intact senses project to cortical brain areas, such as the primary auditory cortex (Heschl’s Gyrus ‒ HG) and secondary auditory cortex (Planum Temporale ‒ PT), which are normally responsible for processing auditory input.^5^

Studies have shown that the cerebellum plays a more active role in speech production and comprehension in deaf individuals compared to normal-hearing individuals. The reason for the increased GM thickness and volume in the cerebellum of deaf individuals has been attributed to this heightened activity.^6^ Lin et al. found a significant relationship between hearing impairment and GM.^7^

There are various studies in the literature that examine in patients with acoustic neuroma, hearing aid users, individuals with bilateral SNHL, or those with unilateral sudden HL. Volumetric changes occur in cerebral and cerebellar structures in individuals with long-term SNHL.^3^, ^6^ In our study, we aimed to compare the normal-hearing side of patients with USNHL and the HL group with a control group using the VBM method to evaluate cerebral and cerebellar gray matter volume and cortical thickness.

## Methods

Between 2023 and 2024, 12 patients with a history of USNHL for more than one year who visited the Ear, Nose, and Throat outpatient clinic at Alanya Training and Research Hospital were included in this study. Twelve individuals with normal hearing, who had brain MRIs taken for other reasons, were selected as the control group.

All control subjects had normal otoscopic findings of the tympanic membrane, and had air-conduction pure-tone thresholds of < 25 dB on audiometry.

### Inclusion criteria

Patients aged of 18 and 65 years with idiopathic, non-progressive USNHL for at least one year, with no underlying secondary etiological factor identified in prior investigations; had an air-bone gap of < 10 dB on PTA, unilateral HL (PTA > 65 dB HL) and normal hearing in the other ear (PTA < 25 dB HL), absence of any known neurological disorder, and no space-occupying lesions in the intracranial or internal acoustic canal on MRI or CT scan.

### Exclusion criteria

Patients with a history of noise exposure, ototoxic drug use, or ear surgery; congenital and/or conductive hearing loss; hearing loss consistent with presbycusis; fluctuating hearing loss, pulsatile tinnitus, vestibular disease, or a history of neurological disease; inflammation in the external or middle ear; contraindications for MRI (e.g., cochlear implants, pacemakers, or prosthetic valves); use of sign language or hearing aids; or a history of psychiatric illness were excluded.

The MRIs of USNHL patients were compared with the images of the normal-hearing side and the control group. Brain MRI examinations were performed using a 1.5 T MRI machine (GE, SIGNA Explorer, General Electric, Milwaukee, USA). Conventional brain MRI sagittal plane T1-weighted (T1A) images were obtained with the 3D Fast Spoiled Gradient Recall acquisition in steady state (3D T1 FSPGR) sequence. The 3D T1 FSPGR sequence was performed with the following parameters: TE = 1.7 msec, TR = 5.95 msec, flip angle = 12°, acquisition matrix = 256 × 256, FOV = 256 mm^2^, number of slices = 170, and slice thickness = 1.0 mm.

The 3D T1 FSPGR sequence images were transferred in DICOM format to a Dell personal computer running a 64-bit Windows 10 operating system. The DICOM format data were converted to niftii format. The segmental volumetric calculation of cerebral-cerebellar structures was performed by uploading the niftii format files to http://volbrain.upv.es via a web browser.

Using the CERES module on the VolBrain site, volumes/cortical thicknesses/gray matter volumes of the cerebellum (right/left/total), lobule I‒II, III, IV, V, VI, VIIB, VIIIA, VIIIB, IX, X, and lobule crus I‒II were calculated.

Additionally, using the Vol2Brain module on the VolBrain site, volumes and thicknesses were calculated in the cerebrum (right/left/total) for the hippocampus, putamen, ventral Diencephalon (Ventral DC), temporal, Fusiform Gyrus (FuG), Planum Polare (PP), Planum Temporale (PT), Inferior Temporal Gyrus (ITG), Middle Temporal Gyrus (MTG), STG, Transverse Temporal Gyrus (TTG), Temporal Pole (TMP), insular, anterior insula (AIns), Posterior Insula (PIns), Central Operculum (CO), FO, and Parietal Operculum (PO).

**Fig. 1:**
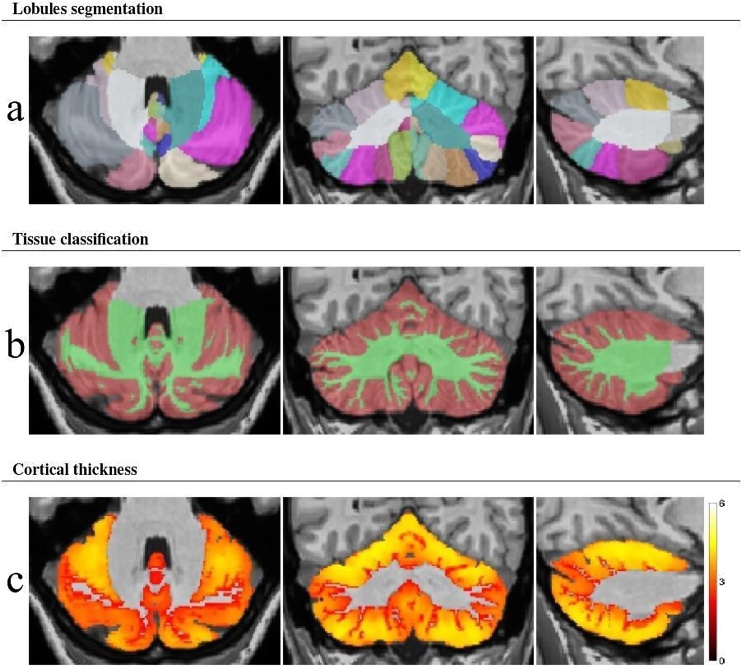
Example images showing cerebellar lobule segmentation (a), gray matter classification (b), and cortical thickness (c).

**Fig. 2:**
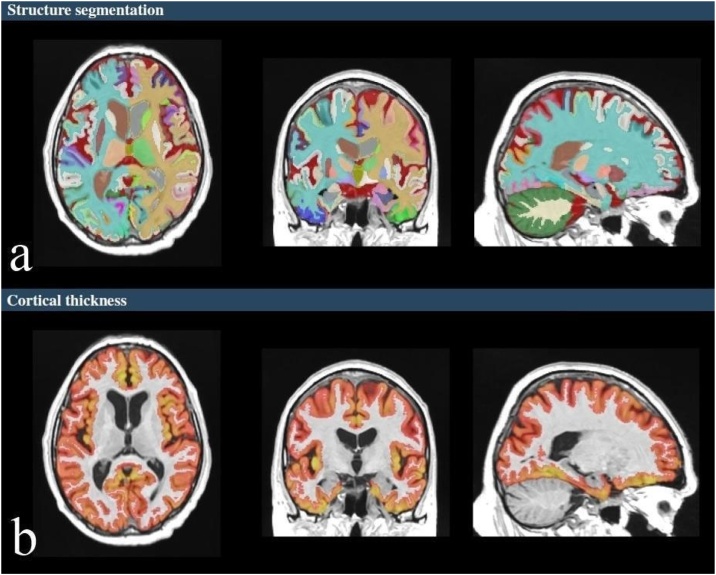
Example of brain structure segmentations (a), segmentation of cortical thickness of brain lobes and gyri (b).

**Fig. 3:**
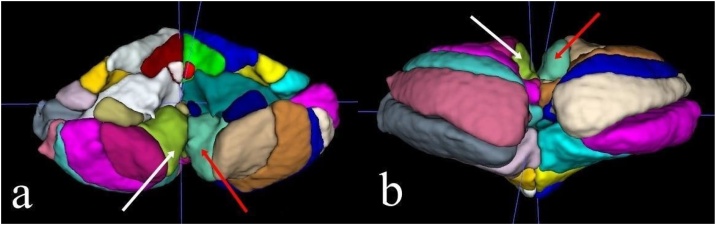
(a‒b) Example images of 3D segmentation created using the ITK-SNAP program with data obtained from VolBrain CERES, showing the right cerebellar lobule IX (white arrows) and left cerebellar lobule IX (red arrows).

Examples of volBrain segmentations of cerebro-cerebellar structures are shown in Fig. 1, Fig. 2, Fig. 3.

The images included in this article are representative examples of the segmentations. In this study, the numerical differences in brain structures exhibiting significant variations were small, making it impossible to visually discern these differences in the images. Therefore, we have only included representative images of the segmentations in the article. Detailed numerical analysis values are presented in Table 1, Table 2.

**Table 1:** Comparison of cerebral data between the hearing loss group and the control group.

	HR ‒ left side	Control left side	*p*-values
Hippocampus volume (cm^3^)	4.02 ± 0.42	4.21 ± 0.51	0.330^a^
Putamen volume (cm^3^)	3.97 ± 0.73	4.45 ± 0.46	0.072^a^
Ventral DC volume (cm^3^)	4.77 ± 0.51	4.81 ± 0.44	0.832^a^
Temporal volume (cm^3^)	52.50 ± 6.89	54.27 ± 5.31	0.486^a^
FuG volume (cm^3^)	7.85 (6.82‒10.33)	8.22 (6.65‒9.94)	0.843^b^
PP volume (cm^3^)	1.67 (1.26‒2.69)	1.58 (1.38‒2.19)	0.514^b^
PT volume (cm^3^)	1.87 ± 0.49	1.71 ± 0.48	0.428^a^
ITG volume (cm^3^)	12.63 ± 1.88	12.81 ± 1.15	0.775^a^
MTG volume (cm^3^)	11.02 ± 2.05	12.51 ± 2.47	0.122^a^
STG volume (cm^3^)	5.97 ± 1.10	5.93 ± 1.06	0.929^a^
TTG volume (cm^3^)	1.63 ± 0.47	1.63 ± 0.36	0.977^a^
TMP volume (cm^3^)	9.20 ± 2.23	9.79 ± 1.45	0.454^a^
Insular volume (cm^3^)	14.55 ± 2.51	14.01 ± 0.73	0.491^a^
AIns volume (cm^3^)	4.20 ± 0.77	4.41 ± 0.34	0.394^a^
PIns volume (cm^3^)	2.35 (1.80‒2.93)	2.20 (2.01‒2.83)	0.514^b^
CO volume (cm^3^)	3.75 ± 0.76	3.74 ± 0.25	0.974^a^
FO volume (cm^3^)	2.09 ± 0.47	1.72 ± 0.18	**0.024**^a^
PO volume (cm^3^)	2.15 ± 0.62	1.88 ± 0.34	0.209^a^
Temporal thickness (mm)	3.27 ± 0.27	3.24 ± 0.16	0.783^a^
FuG thickness (mm)	3.95 ± 0.26	3.88 ± 0.17	0.444^a^
PP thickness (mm)	1.60 ± 0.48	1.31 ± 0.37	0.117^a^
PT thickness (mm)	1.88 ± 0.56	1.53 ± 0.42	0.098^a^
ITG thickness (mm)	3.57 ± 0.26	3.66 ± 0.17	0.337^a^
MTG thickness (mm)	3.07 ± 0.30	3.09 ± 0.26	0.927^a^
STG thickness (mm)	2.66 ± 0.44	2.31 ± 0.36	**0.041**^a^
TTG thickness (mm)	1.86 ± 0.72	1.75 ± 0.44	0.645^a^
TMP thickness (mm)	3.62 ± 0.55	3.79 ± 0.28	0.349^a^
Insular thickness (mm)	2.98 ± 0.46	2.87 ± 0.31	0.499^a^
AIns thickness (mm)	3.85 (2.84‒4.11)	3.78 (3.32‒3.96)	0.671^b^
PIns thickness (mm)	3.39 ± 0.45	3.31 ± 0.41	0.653^a^
CO thickness (mm)	2.44 ± 0.63	2.30 ± 0.44	0.540^a^
FO thickness (mm)	2.76 ± 0.70	2.39 ± 0.38	0.126^a^
PO thickness (mm)	2.17 ± 0.59	1.95 ± 0.40	7

HR, Hearing Loss; DC, Diencephalon; FuG, Fusiform Gyrus; PP, Planum Polare; PT, Planum Temporale; ITG, Inferior Temporal Gyrus; MTG, Middle Temporal Gyrus; STG, Superior Temporal Gyrus; TTG, Transverse Temporal Gyrus; TMP, Temporal Pole; Aıns, Anterior Insula; Pıns, Posterior Insula; CO, Central Operculum; FO, Frontal Operculum; PO, Parietal Operculum.

a Independent Samples *t*-test.

b Mann-Whitney *U* test, data are presented as median (minimum‒maximum) values.

**Table 2:** Comparison of cerebellar data between the hearing loss group and the control group.

	HR ‒ right side	Control right side	p-values
	Mean ± SD Deviation		
Cerebellum (cm^3^)	62.7 ± 8.8	61.9 ± 4.6	0.798^a^
I‒II (cm^3^)	0.051 (0.040‒0.094)	0.051 (0.029‒0.067)	0.755^b^
III (cm^3^)	0.573 ± 0.104	0.699 ± 0.193	0.059^a^
IV (cm^3^)	2.2 (1.6‒3.2)	2.1 (2.1‒3.6)	0.551^b^
V (cm^3^)	3.8 ± 0.8	4.2 ± 0.4	0.130^a^
VI (cm^3^)	8.9 ± 1.3	8.4 ± 0.9	0.253^a^
Crus I (cm^3^)	12.6 (9.9‒18.7)	11.9 (10.5‒13.9)	0.755^b^
Crus II (cm^3^)	8.1 ± 1.4	7.9 ± 1.2	0.710^a^
VIIB (cm^3^)	4.3 (3.6‒7.1)	4.3 (3.5‒5.2)	0.478^b^
VIIIA (cm^3^)	5.7 (4.8‒7.4)	5.7 (4.2‒7.5)	0.977^b^
VIIIB (cm^3^)	3.9 (3.2‒5.4)	3.7 (3.1‒5.7)	0.843^b^
IX (cm^3^)	3.6 ± 0.6	3.3 ± 0.5	0.093^a^
X (cm^3^)	0.63 ± 0.10	0.63 ± 0.07	0.947^a^
Cerebellum cortical thickness	4.54 ± 0.18	4.63 ± 0.18	0.266^a^
Cerebellum cortical thickness norm.	4.07 ± 0.22	4.14 ± 0.13	0.404^a^
I‒II cortical thickness	2.37 ± 0.49	2.21 ± 0.50	0.434^a^
I‒II cortical thickness norm.	2.12 ± 0.45	1.97 ± 0.44	0.414^a^
III cortical thickness	3.79 ± 0.36	3.74 ± 0.52	0.774^a^
III cortical thickness norm.	3.40 ± 0.36	3.34 ± 0.47	0.743^a^
IV cortical thickness	5.00 ± 0.14	5.02 ± 0.14	0.720^a^
IV cortical thickness norm.	4.48 ± 0.13	4.49 ± 0.14	0.859^a^
V cortical thickness	4.97 ± 0.20	4.94 ± 0.11	0.614^a^
V cortical thickness norm.	4.46 ± 0.16	4.42 ± 0.11	0.509^a^
VI cortical thickness	5.07 (4.67‒5.38)	5.07 (4.69‒5.23)	0.551^b^
VI cortical thickness norm.	4.53 ± 0.19	4.47 ± 0.15	0.375^a^
Crus I cortical thickness	4.29 ± 0.42	4.51 ± 0.33	0.159^a^
Crus I cortical thickness norm.	3.84 ± 0.38	4.04 ± 0.28	0.173^a^
Crus II cortical thickness	3.85 ± 0.47	4.19 ± 0.63	0.155^a^
Crus II cortical thickness norm.	3.47 ± 0.50	3.74 ± 0.53	0.199^a^
VIIB cortical thickness	4.57 (4.30‒4.92)	4.73 (3.63‒4.96)	0.443^b^
VIIB cortical thickness norm.	4.12 ± 0.23	4.14 ± 0.30	0.870^a^
VIIIA cortical thickness	4.73 ± 0.14	4.68 ± 0.19	0.446^a^
VIIIA cortical thickness norm.	4.24 ± 0.18	4.19 ± 0.17	0.443^a^
VIIIB cortical thickness	4.94 (3.99‒5.33)	4.92 (4.65‒5.11)	0.843^b^
VIIIB cortical thickness norm.	4.45 (3.47‒4.69)	4.40 (4.16‒4.59)	0.478^b^
IX cortical thickness	4.61 (3.32‒4.95)	4.41 (3.75‒4.89)	0.630^b^
IX cortical thickness norm.	3.96 ± 0.54	3.92 ± 0.33	0.853^a^
X cortical thickness	2.57 ± 0.54	2.58 ± 0.32	0.933^a^
X cortical thickness norm.	2.30 ± 0.49	2.31 ± 0.30	0.950^a^
Cerebellum grey matter (cm^3^)	46.4 ± 6.5	46.0 ± 3.5	0.850^a^
I‒II grey matter (cm^3^)	0.035 (0.029‒0.060)	0.033 (0.025‒0.039)	0.266^b^
III grey matter (cm^3^)	0.47 ± 0.09	0.55 ± 0.16	0.170^a^
IV grey matter (cm^3^)	1.96 ± 0.44	2.13 ± 0.40	0.313^a^
V grey matter (cm^3^)	3.27 ± 0.69	3.59 ± 0.32	0.156^a^
VI grey matter (cm^3^)	8.0 ± 1.1	7.5 ± 0.9	0.233^a^
Crus I grey matter (cm^3^)	10.0 ± 2.3	10.2 ± 1.0	0.882^a^
Crus II grey matter (cm^3^)	6.3 ± 0.9	6.4 ± 1.0	0.756^a^
VIIB grey matter (cm^3^)	3.8 (3.1‒5.7)	3.7 (3.0‒4.6)	0.590^b^
VIIIA grey matter (cm^3^)	4.7 (4.2‒6.6)	4.8 (3.4‒6.5)	0.799^b^
VIIIB grey matter (cm^3^)	3.5 (2.8‒4.9)	3.4 (2.6‒4.8)	0.755^b^
IX grey matter (cm^3^)	3.1 ± 0.4	2.7 ± 0.4	**0.025**^a^
X grey matter (cm^3^)	0.60 ± 0.10	0.59 ± 0.07	0.737^a^

HR, Hearing Loss.

a Independent Samples *t*-test.

b Mann-Whitney *U* test, data are presented as median (minimum‒maximum) values.

For the numerical values obtained from Volbrain:

1 The hearing-impaired sides of the USNHL group were compared with the same randomly selected side from the control group.
2 The intact sides of the USNHL group were compared with the same randomly selected side from the control group.
3 Total (right/left combined) measurements from the USNHL and control groups were compared.
4 The intact side was compared with the impaired side in the USNHL group, and the data were analyzed statistically.

### Statistical analysis

Descriptive data were presented as numbers and percentages, while measurement data were shown as mean ± standard deviation or median (minimum‒maximum) values, as appropriate. The Chi-Square and Fisher’s exact tests were used for the comparison of categorical data where applicable. The Shapiro-Wilk test and histogram graphs were used to assess the assumption of normal distribution. For the comparison of measurements between groups, the Independent Samples *t*-test was used for normally distributed data, while the Mann-Whitney *U* test was applied for non-normally distributed data.

To assess the correlations between the quantitative data in the study, Pearson correlation coefficient was used for parametric data, while Spearman correlation coefficient was used for non-parametric data.

A *p*-value of < 0.05 was considered statistically significant. Bonferroni correction was applied to the p-values for post hoc analyses. The analyses were conducted using the IBM SPSS 23 statistical software.

Ethical approval was obtained from the Institutional Review Board of Alanya Alaaddin Keykubat University School of Medicine Hospital’s ethics committee. Informed consent was obtained from all participants.

## Results

Each group, both the patient and control groups, consisted of 6 females and 6 males. The average age in the patient and control groups was 49.50 ± 15.6 and 49.4 ± 8.1 years, respectively.

In the HL group, 10 participants were right-handed, and 2 were left-handed. In the control group, all participants were right-handed.

There were no significant differences between the USNHL patient and control groups in terms of age and gender (*p* > 0.05).

The duration of hearing loss ranged from a minimum of 1 year to a maximum of 21 years, with an average of 7.1 ± 5.1 years.

In the USNHL participants, 6 individuals had a PTA between 65‒79 dB, while 6 had a PTA of ≥ 95 dB. USNHL was present in the right ear in 4 patients and in the left ear in 8 patients.

In the USNHL patient group, the mean PTA was 47.9 ± 47.2 dB in the right ear and 65.8 ± 37.2 dB in the left ear. In the control group, PTA was < 25 dB.

The measurements of the demographic and auditory characteristics of all participants are shown in Table 3. Detailed information regarding the evaluation of the subjects is presented in Table 1, Table 2.

1 Cerebral volume differences between patients and normal controls: When comparing the left-side data of the NH group with the USNHL group, the FO GM volume (cm^3^) (*p* = 0.024) and STG thickness (*p* = 0.041) measurements were found to be significantly higher in the USNHL group compared to the control group. No statistically significant relationship was found between the groups for the other parameters examined (Table 1).
2 Cerebellar volume differences between patients and normal controls: When comparing the right-side data of the USNHL group with the control group, the cerebellum IX GM volume (cm^3^) in the USNHL group was found to be significantly higher than in the control group (*p* = 0.025). No statistically significant relationship was found between the groups for the other parameters examined (Table 2).
3 Correlations between hearing level and gray matter volume: When the correlations between PTA values and cerebral and cerebellar Gray Matter (GM) volumes and thicknesses were examined, no statistically significant difference was observed (p > 0.05) (Table 4).

**Table 4:** Comparison of patient-side volumes based on audiology data.

	65‒79 dB Range (Mean ± SD)	95 and Above (Mean ± SD)	*p*-values
Age	48.67 ± 16.08	50.33 ± 16.60	0.863^a^
Duration of hearing loss (years)	7.00 (3.00‒10.00)	6.00 (1.00‒21.00)	0.937^b^
Hippocampus volume (cm^3^)	4.03 ± 0.29	4.12 ± 0.46	0.695^a^
Putamen volume (cm^3^)	3.90 ± 0.71	3.96 ± 0.76	0.895^a^
Ventral DC volume (cm^3^)	4.90 ± 0.46	4.69 ± 0.56	0.496^a^
Temporal volume (cm^3^)	52.99 ± 5.24	54.29 ± 6.14	0.701^a^
FuG volume (cm^3^)	8.29 ± 1.46	8.79 ± 1.67	0.593^a^
PP volume (cm^3^)	1.85 ± 0.44	1.79 ± 0.32	0.773^a^
PT volume (cm^3^)	1.76 ± 0.55	1.74 ± 0.53	0.965^a^
ITG volume (cm^3^)	12.97 ± 1.71	12.22 ± 1.37	0.421^a^
MTG volume (cm^3^)	11.54 ± 2.00	12.04 ± 1.33	0.618^a^
STG volume (cm^3^)	6.39 ± 1.30	6.10 ± 0.29	0.596^a^
TTG volume (cm^3^)	1.40 ± 0.44	1.75 ± 0.45	0.204^a^
TMP volume (cm^3^)	8.79 ± 2.31	9.86 ± 1.86	0.394^a^
Insular volume (cm^3^)	13.94 ± 2.85	14.76 ± 2.18	0.588^a^
AIns volume (cm^3^)	4.00 ± 0.90	4.48 ± 0.66	0.311^a^
PIns volume (cm^3^)	2.33 ± 0.33	2.34 ± 0.34	0.972^a^
CO volume (cm^3^)	3.51 ± 0.89	3.87 ± 0.51	0.405^a^
FO volume (cm^3^)	1.95 ± 0.53	2.08 ± 0.45	0.667^a^
PO volume (cm^3^)	2.15 ± 0.68	1.99 ± 0.69	0.695^a^
Temporal thickness (mm)	3.25 ± 0.24	3.43 ± 0.32	0.316^a^
FuG thickness (mm)	3.93 ± 0.32	3.88 ± 0.25	0.761^a^
PP thickness (mm)	1.60 ± 0.54	1.71 ± 0.50	0.721^a^
PT thickness (mm)	1.68 ± 0.52	2.22 ± 0.51	0.100^a^
ITG thickness (mm)	3.61 ± 0.26	3.72 ± 0.31	0.508^a^
MTG thickness (mm)	3.10 ± 0.20	3.30 ± 0.34	0.238^a^
STG thickness (mm)	2.63 ± 0.49	2.89 ± 0.49	0.386^a^
TTG thickness (mm)	1.73 ± 0.72	2.08 ± 0.67	0.396^a^
TMP thickness (mm)	3.49 ± 0.60	3.89 ± 0.51	0.244^a^
Insular thickness (mm)	2.85 ± 0.48	3.17 ± 0.44	0.257^a^
AIns thickness (mm)	3.52 ± 0.43	3.89 ± 0.38	0.141^a^
PIns thickness (mm)	3.36 ± 0.61	3.36 ± 0.44	0.996^a^
CO thickness (mm)	2.29 ± 0.71	2.71 ± 0.52	0.275^a^
FO thickness (mm)	2.54 ± 0.78	2.98 ± 0.61	0.301^a^
PO thickness (mm)	2.05 ± 0.59	2.31 ± 0.66	0.483^a^

DC, Diencephalon; FuG, Fusiform Gyrus; PP, Planum Polare; PT, Planum Temporale; ITG, Inferior Temporal Gyrus; MTG, Middle Temporal Gyrus; STG, Superior Temporal Gyrus; TTG, Transverse Temporal Gyrus; TMP, Temporal Pole; Aıns, Anterior Insula; Pıns, Posterior Insula; CO, Central Operculum; FO, Frontal Operculum; PO, Parietal Operculum.

a Independent samples *t*-test.

b Mann-Whitney *U* test, data are presented as median (minimum‒maximum) values.
4 Correlations between duration and gray matter volume: When the correlations between duration and cerebral and cerebellar Gray Matter (GM) volumes and thicknesses were examined, no statistically significant difference was observed (*p* > 0.05) (Table 5).

**Table 5:** Correlation between hearing loss duration and the volumes of the affected and unaffected sides.

	Duration of Hearing Loss (Years)			
	Affected Side		Unaffected Side	
	*r*	*p*	*r*	*p*
Hippocampus volume (cm^3^)	−0.011	0.973	−0.140	0.663
Putamen volume (cm^3^)	0.188	0.559	0.223	0.486
Ventral DC volume (cm^3^)	0.214	0.504	0.254	0.425
Temporal volume (cm^3^)	0.163	0.612	0.073	0.822
FuG volume (cm^3^)	0.416	0.178	0.356	0.257
PP volume (cm^3^)	0.164	0.610	0.439	0.153
PT volume (cm^3^)	0.063	0.845	0.346	0.270
ITG volume (cm^3^)	0.174	0.588	0.347	0.270
MTG volume (cm^3^)	−0.146	0.650	−0.350	0.265
STG volume (cm^3^)	−0.044	0.892	0.059	0.855
TTG volume (cm^3^)	0.344	0.274	0.117	0.717
TMP volume (cm^3^)	0.012	0.971	0.034	0.915
Insular volume (cm^3^)	0.112	0.729	0.312	0.323
AIns volume (cm^3^)	0.212	0.508	0.229	0.475
PIns volume (cm^3^)	−0.218	0.496	−0.157	0.626
CO volume (cm^3^)	0.120	0.710	0.276	0.386
FO volume (cm^3^)	0.078	0.810	0.200	0.534
PO volume (cm^3^)	0.082	0.800	0.503	0.096
Temporal thickness (mm)	0.496	0.101	0.172	0.592
FuG thickness (mm)	0.381	0.222	0.312	0.323
PP thickness (mm)	0.142	0.660	0.144	0.655
PT thickness (mm)	0.446	0.146	0.115	0.721
ITG thickness (mm)	0.365	0.243	0.169	0.600
MTG thickness (mm)	0.490	0.106	−0.093	0.773
STG thickness (mm)	0.329	0.296	0.018	0.955
TTG thickness (mm)	0.338	0.283	0.303	0.338
TMP thickness (mm)	0.294	0.353	0.123	0.702
Insular thickness (mm)	0.295	0.352	0.169	0.600
AIns thickness (mm)	0.196	0.541	0.081	0.803
PIns thickness (mm)	−0.051	0.874	0.042	0.896
CO thickness (mm)	0.297	0.349	0.170	0.597
FO thickness (mm)	0.277	0.383	0.210	0.513
PO thickness (mm)	0.461	0.131	0.307	0.331

Statistical test, Pearson correlation analysis; DC, Diencephalon; FuG, Fusiform Gyrus; PP, Planum Polare; PT, Planum Temporale; ITG, Inferior Temporal Gyrus; MTG, Middle Temporal Gyrus; STG, Superior Temporal Gyrus; TTG, Transverse Temporal Gyrus; TMP, Temporal Pole; Aıns, Anterior Insula; Pıns, Posterior Insula; CO, Central Operculum; FO, Frontal Operculum; PO, Parietal Operculum.
5 There was no significant correlation between hearing loss duration and age at all (Rho = −0.389, *p* = 0.212).

**Table 3:** Demographic data and general hearing loss ınformation of the Hearing Loss (HL) group and control group.

	HL Group	Control Group	*p*-values
	n (%)	n (%)	
Gender			>0.999^a^
Female	6 (50.0)	6 (50.0)	
Male	6 (50.0)	6 (50.0)	
Age	49.50 ± 15.61	49.42 ± 8.10	0.987^c^
Dominant Hand			0.478^b^
Right	10 (83.3)	12 (100.0)	
Left	2 (16.7)	0 (0.0)	
Side of Hearing Loss			>0.999^b^
Right	4 (33.3)	4 (33.3)	
Left	8 (66.7)	8 (66.7)	
Audiometry (Right)	47.92 ± 47.20		*
Audiometry (Left)	65.83 ± 37.18		*
Duration of Hearing Loss (years)	7.08 ± 5.09		*

a Chi-Square test.

b Fisher’s exact test.

c Independent Samples *t*-test, data presented as mean ± standard deviation.

## Discussion

In this study, differences in cerebral and cerebellar GM volume and cortical thickness, as well as their relationship with HL, were investigated in patients with long-term unilateral SNHL. Our literature review did not identify any study that analyzed both cerebral and cerebellar structures together using VolBrain. This study focused on the cerebral and cerebellar morphology in adults with long-term USNHL. To exclude the possibility that the morphometric changes were due to tumor growth and to more clearly identify changes associated with hearing loss, USNHL patients with no history of tumors were selected.

Various studies have used the VBM method to identify structural and functional changes in the brains of SNHL patients, reporting volumetric changes in the GM in both auditory and non-auditory areas.^8^ Total cerebral and cerebellar volume changes, differences between the right and left hemispheres, and sub-segment volume changes have been addressed in various studies. Koops et al. identified differences in Gray Matter (GM) volume and thickness in both auditory and non-auditory brain regions in patients with Sensorineural Hearing Loss (SNHL). They particularly reported a correlation between GM volume and age.^9^ However, in our study, no correlation with age was observed. In the SNHL group without tinnitus, a reduction in GM volume was observed in the Middle Temporal Gyrus (MTG) and Inferior Temporal Gyrus (ITG) compared to the healthy group, whereas no difference was found in the tinnitus group.^9^ In our study, all SNHL patients had accompanying tinnitus, and no significant differences in volume or thickness were observed in the MTG and ITG regions.

In a study by Smith et al. on deaf and normal-hearing pediatric patients, no volume difference was observed between the right and left sides of the auditory cortex. However, when White Matter (WM) and GM subgroups were analyzed, an increase in GM volume was found in the SNHL group compared to the normal-hearing group.^13^ In our study, when the temporal lobe volumes, including the auditory cortex, were compared, no significant differences were observed between the SNHL and healthy groups or between the hearing-impaired and intact sides within the SNHL group.

In a study by Ren et al. on patients with presbycusis, a decrease in GM volume, including in the insula and auditory cortex, was reported compared to healthy individuals.^10^ Similarly, Manno et al. reported GM volume reductions in the frontal lobe and insula. It has been suggested that differences in auditory inputs, age groups, acquired or congenital nature of hearing loss, and compensatory mechanisms may result in region-specific GM changes (increases or decreases). Additionally, studies have examined the PT region.^11^ In our study, insula and PT were analyzed, and no statistically significant differences in GM volume or thickness were found. Unlike other studies, none of the patients in our study had a diagnosis of presbycusis.

Automatic brain volume analysis is a relatively new technology. Automatic brain segmentation systems have been shown to provide more accurate results than traditional manual segmentations.^12^ Volbrain, a web-based automatic brain segmentation program, is a novel application. Romero et al. reported that Volbrain produced more accurate results compared to other brain segmentation methods, such as SUIT, MAGeT, and RASCAL.^13^

Volbrain is based on multi-atlas label fusion technology, providing fast and accurate volumetric information within minutes, with systematic error correction.^14^

Different volumetric studies have yielded various results, and a clear consensus has not been reached. Some studies have reported preserved GM volume in the auditory cortex,^15^ others have found increased GM volume,^16^ while some have reported a decrease in GM volume.^8^ According to the theory of intermodal plasticity, neurons in the auditory cortex do not degenerate even in the absence of auditory input, as they can respond to other sensory inputs, such as visual and tactile signals. Therefore, no GM volume loss has been observed in the early stages of SNHL.^17^ As SNHL progresses, neurons in the auditory area gradually degenerate due to prolonged auditory deprivation, consistent with Darwin’s law of use and disuse. In fact, the GM volumes of these brain regions are negatively correlated with the duration of the disease.^12^

In our study, when the correlations between the cerebral volume and thickness of the hearing-impaired and healthy sides and disease duration were examined, no statistically significant relationship was found among the analyzed parameters (Table 5). Some researchers have observed that there is no significant change in GM volume in the auditory areas of deaf patients but noted structural changes in WM in the early stages of the disease.^15^, ^18^ In our study, no statistically significant relationship was found between the total volume measurements of the USNHL and control groups.

The HG is a major component of the posterior part of the STG and forms the anatomical substrate of the primary auditory area. The STG plays a crucial role in auditory perception.^14^ GM volume reduction in primary sensory areas is significantly related to subjective hearing ability, while the duration of hearing loss is associated with decreased GM volumes in cortical areas involved in higher-order cognitive processing. These findings suggest that the severity and duration of UHL contribute to GM volume changes in auditory and higher-order functional structures, respectively.^6^ A study on SNHL infants demonstrated increased GM volume in the anterior HG and STG.^15^ In Shibata’s study, GM volume was found to be similar between deaf and NH individuals.^19^

Hribar et al. compared the structural brain characteristics of congenitally deaf and normal-hearing adults, observing a significant reduction in left HG WM volume in the deaf group, with no statistically significant change in HG GM volume between the groups. They reported that GM volume was preserved in the auditory areas, with no change in surface area or thickness. In deaf individuals, there was no difference between groups in GM volume, thickness, or surface area of the auditory GM.^20^ GM volume in the frontal region was shown to decrease with HL, while GM volume in the temporal cortex increased.^13^ Husain et al. found that patients with bilateral hearing loss had reduced GM volume around the auditory cortex and disrupted WM microstructural integrity.^14^ Increased cortical thickness in the bilateral STG has been observed in deaf adults compared to normal-hearing adults, with these morphological changes in key brain regions attributed to compensatory cross-modal reorganization.^21^

In our study, no statistically significant difference was found in STG GM volume between the UHL and NH sides or between the SNHL and control groups. However, STG thickness measurements were significantly higher in the hearing-impaired group, supporting the cross-modal theory.

The FO is associated with the prefrontal association cortex, playing a role in thought, cognition, and planning behavior, and is connected to the temporal region.^16^ It exhibits left hemisphere dominance.^22^ A connection has been shown between the FO and the sensorimotor cortex.^23^ In our study, when comparing the left-side data between the hearing loss and control groups, FO volume measurements were significantly higher in the hearing loss group than in the control group. This increase suggests a neuroadaptive mechanism resulting from patients having only one auditory organ.

According to Van Essen’s theory, cortical folding is the result of axonal tension between interconnected regions; the mirror-symmetric tonotopic organization of HG may explain its shape, and this organization may also explain the increased cortical thickness and preserved total HG GM volume in the deaf.^24^ The lack of change in HG GM volume in our study supports this theory.

Findings related to the neuroplasticity of individuals with varying levels of UHL demonstrate the critical role of auditory input in GM volume, particularly in the temporal lobe. Wang X et al. conducted VBM studies on patients with hearing loss due to acoustic neuroma. Since tumor growth in the cerebellopontine angle may alter anatomical relationships in the brain, it has been suggested that morphological studies focus on UHL patients without growing brain tumors.^6^ Studies on UHL patients have reported a correlation between the severity of hearing loss and decreased GM volume in the temporal lobe, indicating that peripheral hearing ability plays a role in reducing GM volume in the auditory cortex.

In our study, when the correlations between the cerebral volume and thickness of the hearing-impaired and healthy sides and the severity of hearing loss were examined, no statistically significant correlation was found among the analyzed parameters (Table 4). There is no clear consensus regarding the duration of long-term HL. In their study, Xu et al. defined long-term HL as lasting more than one year, but different studies have adopted different time frames.^1^, ^25^, ^26^ In our study, we included patients who had complaints lasting longer than one year.

There is strong evidence that the cerebellum, particularly the vestibulocerebellum, makes vital contributions to self-motion perception. It has long been known that lesions in Larsell’s lobules X and IX alter the temporal and three-dimensional spatial processing of vestibular information.^27^ Different studies have demonstrated varying morphological changes in cerebellar GM structure. Hribar et al. found that neurons did not significantly degenerate in the absence of auditory input, with increased GM volume in the bilateral cerebellum and a larger cerebellum compared to normal-hearing individuals.^15^ Conversely, Olulade et al. reported a decrease in cerebellar GM.^8^ Rongmiao et al. demonstrated a reduction in GM volume in the cerebellum of deaf individuals.^17^ In Hribar et al.’s study, cerebellar GM thickness changes were not statistically significant; however, they reported an increase in cerebellar GM volume in early deafness.^20^ In USNHL patients, increased connections with some brain regions and decreased connections with others in the cerebellum have been observed, suggesting a link with the duration of hearing loss.^25^ Most previous studies have focused on individuals who were completely deaf, and these volumetric studies have shown that GM volume in the auditory cortex was either preserved or decreased in deaf individuals.^19^ It has been suggested that the preservation of GM may be due to cross-modal plasticity as well as the lack of sensitivity of the methods used to detect microstructural changes in GM.^28^

In our study, when comparing the right-side data of the USNHL group with the control group, cerebellum IX GM volume was significantly higher in the hearing loss group than in the control group. These findings support the studies showing increased cerebellum GM volume and suggest that this may be due to compensatory structural reorganization in response to hearing loss.

### Limitations

This study has several limitations. Most notably, the sample size was insufficient. The patient's speech comprehension and psychiatric evaluations were not conducted. Additionally, not all participants were right-handed, which was considered as a factor that might influence hemispheric dominance. If more cases can be collected, we plan to conduct a more detailed differentiation of the cases and perform an advanced study.

## Conclusion

Our study is the first to use Volbrain to investigate hearing loss that is unilateral, untreated with hearing devices, not consistent with presbycusis, and not related to tumors or identifiable etiological factors. In examining, statistically significant differences were found between the USNHL group and the control group in the FO and STG regions, as well as in lobule IX, which are involved in functions related to perception, cognition, and the temporal and three-dimensional spatial processing of vestibular information. Clinically, we recommend that patients with disorders related to perceptual, cognitive, temporal, and three-dimensional spatial processing should also be evaluated for USNHL. When individuals with hearing loss are compared to their normal-hearing side, no significant differences were found; however, comparing them to a healthy control group revealed morphological changes. Knowing that compensatory neuroadaptive changes may occur in cerebral and cerebellar structures when comparing patients to controls could be useful in interpreting these cases. Nevertheless, to eliminate potential inconsistencies due to the small sample size, we recommend conducting larger-scale studies with a greater number of participants and comparing Volbrain with other segmentation programs.

## Declaration of competing interest

The authors declare no conflicts of interest.
